# Supplementary material for: Content-rich biological network constructed by mining PubMed abstracts
Source: BMC Bioinformatics. 2004 Oct 8;5:147. doi: 10.1186/1471-2105-5-147 (PMC528731; doi:10.1186/1471-2105-5-147)
Supplement: Additional File 2 — The original results of the above study (non-essential files are deleted to keep the file size under the limit set by BMC bioinformatics). [file 1471-2105-5-147-S2.bz2 › chilibotAdditionalFile2/dip30/227ID7954478E744/html/CTNNB1_CTNNA1.html]

 


 **CTNNB1** and **CTNNA1** 
  
Found 3187 abstracts in PubMed, retrieved 30.  
 

 What does Google say? 
 PDF only 
| .edu only 

---

**Interactive relationship** (e.g. stimulation, inhibition, etc)

**Inhibitory relationship**- We isolated the gene HMG2L1 and showed that injection of Xenopus HMG2L1 xHMG2L1 mRNA into Xenopus embryos inhibited Wnt beta catenin  [ **CTNNA1** ]   [ **CTNNB1** ]  induced axis duplication and expression of Wnt beta catenin  [ **CTNNA1** ]   [ **CTNNB1** ]  target genes.  Ref: 12875653 Genes CellsGenes Cells,
**Neutral relationship**- These results suggest that interactions of the N terminal domain of beta catenin  [ **CTNNA1** ]   [ **CTNNB1** ]  with alpha catenin  [ **CTNNA1** ]   [ **CTNNB1** ]  and of the C terminal domain with PDZ domain containing proteins are required, respectively, to initiate and shape axon arbors of RGCs in vivo.  Ref: 12878698 J NeurosciJ Neurosci,
- By using a panel of deletion mutants of both proteins we demonstrate that the interaction is mediated by the PDZ domain of TIP 1 and requires primarily the last four amino acids of beta catenin  [ **CTNNA1** ]   [ **CTNNB1** ] .  Ref: 12874278 J Biol ChemJ Biol Chem,
- Wnt beta catenin  [ **CTNNA1** ]   [ **CTNNB1** ]  signalling induces the stabilization of cytosolic beta catenin  [ **CTNNA1** ]   [ **CTNNB1** ] , which interacts with TCF LEF 1 transcription factors, thereby inducing expression of Wnt target genes.  Ref: 12875653 Genes CellsGenes Cells,

**Non-interactive relationship** (e.g. studied together, co-existance, homology, etc.)

- These mutations lead to nuclear localization of beta catenin  [ **CTNNA1** ]   [ **CTNNB1** ] , which can be demonstrated immunohistochemically.  Ref: 12877730 Histopathology, 2003
- Expression of E cadherin, alpha catenin  [ **CTNNA1** ]   [ **CTNNB1** ] , and beta catenin  [ **CTNNA1** ]   [ **CTNNB1** ]  in the process of lymph node metastasis in oral squamous cell carcinoma.  Ref: 12888830 Br J Cancer, 2003
- CONCLUSIONS The cadherin catenin  [ **CTNNA1** ]   [ **CTNNB1** ]  complex is a specific marker of Bowman s epithelial cells in human glomeruli.  Ref: 12877733 HistopathologyHistopathology, 2003
- Differentiation of ovarian mucinous carcinoma and metastatic colorectal adenocarcinoma by immunostaining with beta catenin  [ **CTNNA1** ]   [ **CTNNB1** ] .  Ref: 12877730 Histopathology, 2003
- Src activity was increased in QLalpha12 MDCK cells as assessed by Src autophosphorylation, and beta catenin  [ **CTNNA1** ]   [ **CTNNB1** ]  tyrosine phosphorylation was also increased, although there was no detectable increase in Rho activity.  Ref: 12890651 Am J Physiol Cell PhysiolAm J Physiol Cell Physiol, 2003
- the latter suppresses beta catenin  [ **CTNNA1** ]   [ **CTNNB1** ]  by promoting its degradation through enhanced inactivation of glycogen synthase kinase 3 GSK3.  Ref: 12883684 Oncol RepOncol Rep, 2003
- For comparison, 40 non mucinous carcinomas of the ovary and 42 metastatic adenocarcinomas from other organs were also immunostained with antibodies against beta catenin  [ **CTNNA1** ]   [ **CTNNB1** ] .  Ref: 12877730 Histopathology, 2003
- In this study we used liver neoplasms induced by several chemical carcinogens to investigate potential nuclear targets associated with beta catenin  [ **CTNNA1** ]   [ **CTNNB1** ]  Wnt signaling and potential membrane associated beta catenin  [ **CTNNA1** ]   [ **CTNNB1** ]  binding partners.  Ref: 12878043 Toxicol Appl Pharmacol, 2003
- Low to high gamma catenin  [ **CTNNA1** ]   [ **CTNNB1** ]  and ezrin expression was seen in cytoplasm of blastemal and epithelial cells of primary and metastatic tumours.  Ref: 12890743 Mol Pathol, 2003
- Additional analysis revealed that the axons of RGCs that did not form arbors after overexpression of DeltaARM or the N terminal domain of beta catenin  [ **CTNNA1** ]   [ **CTNNB1** ]  were frequently mistargeted within the tectum.  Ref: 12878698 J NeurosciJ Neurosci, 2003
- beta Catenin  [ **CTNNA1** ]   [ **CTNNB1** ]  is an executor of Wnt signaling and it can control cell fate and specification.  Ref: 12881717 Oncogene, 2003
- These data demonstrate a direct role of E cadherin catenin  [ **CTNNA1** ]   [ **CTNNB1** ]  complex organization in the regulation of MMPs and suggest an implication of this regulation in the expression of an invasive phenotype by bronchial tumor cells.  Ref: 12875984 Am J Pathol, 2003
- Myogenic signaling by lithium in cardiomyoblasts is Akt independent but requires activation of the beta catenin  [ **CTNNA1** ]   [ **CTNNB1** ]  Tcf Lef pathway.  Ref: 12878481 J Mol Cell Cardiol, 2003
- p120 catenin  [ **CTNNA1** ]   [ **CTNNB1** ]  p120 was originally identified as a tyrosine kinase substrate, and subsequently shown to regulate cadherin mediated cell cell adhesion.  Ref: 12885254 Biochemistry, 2003
- Here we show that beta catenin  [ **CTNNA1** ]   [ **CTNNB1** ]  signaling is required for the formation of the distal, but not the proximal, airways.  Ref: 12885771 J Biol ChemJ Biol Chem, 2003
- The axons of RGCs overexpressing the N terminal domain of beta catenin  [ **CTNNA1** ]   [ **CTNNB1** ]  developed no or very few branches.  Ref: 12878698 J NeurosciJ Neurosci, 2003
- These results are consistent with a signaling pathway initiated by structural changes in the adherens junction in which adherens junction derived plakoglobin regulates nuclear transcription by antagonizing the binding of beta catenin  [ **CTNNA1** ]   [ **CTNNB1** ]  to TCF LEF proteins.  Ref: 12880414 J Invest Dermatol, 2003
- We have studied the effects of the organophosphorous pesticide malathion on cell viability, actin cytoskeleton, cell adhesion complex E cadherin beta catenin  [ **CTNNA1** ]   [ **CTNNB1** ] , and Rho and Rac1 GTPases from the human mammary carcinoma cell line MCF 7.  Ref: 12888906 Int J Oncol, 2003
- As shown by immunoprecipitation, the re expressed E cadherin was able to sequestrate one part of free cytoplasmic beta catenin  [ **CTNNA1** ]   [ **CTNNB1** ]  in BZR cells.  Ref: 12875984 Am J Pathol, 2003
- To investigate the existence of adherens junction mediated signaling, we used peroxovanadate to tyrosine phosphorylate plakoglobin and beta catenin  [ **CTNNA1** ]   [ **CTNNB1** ]  and to dissociate adherens junctions.  Ref: 12880414 J Invest Dermatol, 2003
- During apoptosis, adherens junctions, including VE cadherin and beta and gamma catenin  [ **CTNNA1** ]   [ **CTNNB1** ]  were cleaved and alpha catenin  [ **CTNNA1** ]   [ **CTNNB1** ]  was decreased.  Ref: 12878166 Exp Cell Res, 2003
- CONCLUSIONS Our findings indicate that xHMG2L1 may negatively regulate Wnt beta catenin  [ **CTNNA1** ]   [ **CTNNB1** ]  signalling,.  Ref: 12875653 Genes CellsGenes Cells, 2003
- ... axons of RGCs overexpressing the C terminal domain of beta catenin  [ **CTNNA1** ]   [ **CTNNB1** ]  formed arbors with long, tangled branches.  Ref: 12878698 J NeurosciJ Neurosci, 2003
- METHODS Immunohistochemistry was used to determine the expression and cellular distribution of E cadherin, beta catenin  [ **CTNNA1** ]   [ **CTNNB1** ] , gamma catenin  [ **CTNNA1** ]   [ **CTNNB1** ] , and ezrin in primary and metastatic Wilms s tumours.  Ref: 12890743 Mol Pathol, 2003
- Nuclear translocation was associated with increased plakoglobin and decreased beta catenin  [ **CTNNA1** ]   [ **CTNNB1** ]  binding to nuclear TCF LEF and downregulation of gene transcription from TCF LEF reporter constructs.  Ref: 12880414 J Invest Dermatol, 2003
- Deletion of exon 3 from the endogenous beta catenin  [ **CTNNA1** ]   [ **CTNNB1** ]  gene in differentiating mammary alveolar epithelium of the mouse results in the generation of an activated protein that lacks amino acids 5 80.  Ref: 12881717 Oncogene, 2003
- Because changes in cell cell adhesion have profound effects on cellular behavior, we hypothesized a link between the adhesion and signaling functions of plakoglobin and beta catenin  [ **CTNNA1** ]   [ **CTNNB1** ] .  Ref: 12880414 J Invest Dermatol, 2003
- To further understand the molecular process of transdifferentiation, the expression of genes in mammary tissue was profiled in the absence and presence of activated of beta catenin  [ **CTNNA1** ]   [ **CTNNB1** ] .  Ref: 12881717 Oncogene, 2003
- Significantly greater reduction in expression levels of E cadherin, alpha catenin  [ **CTNNA1** ]   [ **CTNNB1** ]  and beta catenin  [ **CTNNA1** ]   [ **CTNNB1** ]  was found in the metastatic group n=64 compared to the nonmetastatic group n=95 P=0.00.  Ref: 12888830 Br J Cancer, 2003
- These data suggest that TIP 1 may represent a novel regulatory element in the wnt beta catenin  [ **CTNNA1** ]   [ **CTNNB1** ]  signaling pathway.  Ref: 12874278 J Biol ChemJ Biol Chem, 2003
- Once activated, beta catenin  [ **CTNNA1** ]   [ **CTNNB1** ]  then interacts with the Lef Tcf complex to regulate expression of myogenic inducing genes.  Ref: 12878481 J Mol Cell Cardiol, 2003
- The PDZ protein TIP 1 inhibits beta catenin  [ **CTNNA1** ]   [ **CTNNB1** ]  transcriptional activity and growth of colorectal cancer cells.  Ref: 12874278 J Biol ChemJ Biol Chem, 2003
- Expression of potential beta catenin  [ **CTNNA1** ]   [ **CTNNB1** ]  targets, cyclin D1, c Jun, c Myc, E cadherin, and EGFR in chemically induced hepatocellular neoplasms from B6C3F1 mice.  Ref: 12878043 Toxicol Appl Pharmacol, 2003
- TIP 1 overexpression resulted in a dose dependent decrease in the transcriptional activity of beta catenin  [ **CTNNA1** ]   [ **CTNNB1** ]  when tested on the TOP FOPFLASH reporter system.  Ref: 12874278 J Biol ChemJ Biol Chem, 2003
- Coimmunoprecipitation studies from nuclear fractions, gel shift assays, and transient transfections with T cell factor TCF lymphoid enhancer factor LEF optimized promoter reporter constructs were used to investigate the ability of plakoglobin and beta catenin  [ **CTNNA1** ]   [ **CTNNB1** ]  that had redistributed from the membrane to the nucleus to form functional transcriptional regulatory complexes with TCF LEF family member transcription factors.  Ref: 12880414 J Invest Dermatol, 2003
- Prognosis did not correlate with the nuclear beta catenin  [ **CTNNA1** ]   [ **CTNNB1** ]  expression.  Ref: 12883492 HepatologyHepatology, 2003
- beta catenin  [ **CTNNA1** ]   [ **CTNNB1** ]  is required for specification of proximal distal cell fate during lung morphogenesis.  Ref: 12885771 J Biol ChemJ Biol Chem, 2003
- Tyrosine phosphorylation of plakoglobin and beta catenin  [ **CTNNA1** ]   [ **CTNNB1** ]  resulted in their rapid translocation from the cell membrane to the nucleus.  Ref: 12880414 J Invest Dermatol, 2003
- This is supported by the finding that beta catenin  [ **CTNNA1** ]   [ **CTNNB1** ]  known to be overexpressed in JAs acts as a co activator of the AR.  Ref: 12883689 Oncol RepOncol Rep, 2003
- Accumulating evidence has highlighted the importance of beta catenin  [ **CTNNA1** ]   [ **CTNNB1** ]  mediated activation of T cell factor TCF lymphoid enhancer factor LEF transcription during thymocyte development.  Ref: 12884861 Eur J Immunol, 2003
- Changes in apoptosis were monitored in cell lines engineered to express stabilising mutations in beta catenin  [ **CTNNA1** ]   [ **CTNNB1** ] .Findings TCF beta catenin  [ **CTNNA1** ]   [ **CTNNB1** ]  stimulated a six fold to 12 fold increased expression of the survivin gene in colorectal cancer cells.  Ref: 12885482 Lancet, 2003
- We investigated immunohistochemical expression of E cadherin, alpha catenin  [ **CTNNA1** ]   [ **CTNNB1** ]  and beta catenin  [ **CTNNA1** ]   [ **CTNNB1** ]  in 159 tissue samples from patients with oral squamous cell carcinoma and examined the correlation between their expressions and the presence of regional lymph node metastasis.  Ref: 12888830 Br J Cancer, 2003
- When the gene for beta catenin  [ **CTNNA1** ]   [ **CTNNB1** ]  was conditionally excised in epithelial cells of the developing mouse lung prior to E14.5, the proximal lung tubules grew and differentiated appropriately.  Ref: 12885771 J Biol ChemJ Biol Chem, 2003
- The in vivo complex formation between beta catenin  [ **CTNNA1** ]   [ **CTNNB1** ]  and TIP 1 was verified by coimmunoprecipitation and a direct physical association was revealed by GST pull down experiments in vitro.  Ref: 12874278 J Biol ChemJ Biol Chem, 2003
- A key component of the pathway is beta catenin  [ **CTNNA1** ]   [ **CTNNB1** ]  that, in association with TCF 4, directly regulates the expression of wnt responsive genes.  Ref: 12874278 J Biol ChemJ Biol Chem, 2003
- Immunocharacterization with beta catenin  [ **CTNNA1** ]   [ **CTNNB1** ] , E cadherin, cytokeratins AE1 AE3, epithelial membrane antigen, S 100 protein, smooth muscle actin, and vimentin was also performed.  Ref: 12883237 Am J Surg Pathol, 2003
- BACKGROUND The E cadherin catenin  [ **CTNNA1** ]   [ **CTNNB1** ]  adhesion complex is crucial for intercellular adhesiveness and maintenance of tissue architecture.  Ref: 12890743 Mol Pathol, 2003
- beta Catenin  [ **CTNNA1** ]   [ **CTNNB1** ]  mutations in liver tumors induced by 2 amino dimethylimidazo f quinoline in CDF 1 mice.  Ref: 12893427 Cancer Lett, 2003
- Adhesion Associated and PKC Modulated Changes in Serine Threonine Phosphorylation of p120 Catenin  [ **CTNNA1** ]   [ **CTNNB1** ] .  Ref: 12885254 Biochemistry, 2003
- Background Colorectal cancer is thought to originate in the expansion of colonic crypt cells as a result of aberrant gene expression caused by transcription factors of the T cell factor TCF beta catenin  [ **CTNNA1** ]   [ **CTNNB1** ]  family.  Ref: 12885482 Lancet, 2003
- Strong expression of cyclin D1, in a pattern similar to that observed previously for beta catenin  [ **CTNNA1** ]   [ **CTNNB1** ] , was observed by Western analysis for all five hepatoblastomas examined regardless of treatment.  Ref: 12878043 Toxicol Appl Pharmacol, 2003
- These data indicate that except for the occasional disruption of the Wnt pathway through beta catenin  [ **CTNNA1** ]   [ **CTNNB1** ]  mutations, the genetic pathways involved in the development of HCC differ significantly between human liver cancer and tumors induced in mice by MeIQ, but do not rule out the possibility that heterocyclic amines constitute a carcinogenic risk factor in humans.  Ref: 12893427 Cancer Lett, 2003
- This was accompanied with a diffuse beta catenin  [ **CTNNA1** ]   [ **CTNNB1** ]  staining in the cytoplasm and a decreased F actin stress fibers in some rounded cells.  Ref: 12878166 Exp Cell Res, 2003
- VE cadherin and beta catenin  [ **CTNNA1** ]   [ **CTNNB1** ]  at cell periphery were decreased and the discontinuity in alignment was found as observed with immunofluorescence microscopy.  Ref: 12878166 Exp Cell Res, 2003
- SSCP followed by direct DNA sequencing revealed mutations in exon 2 of the beta catenin  [ **CTNNA1** ]   [ **CTNNB1** ]  gene in 2 of 16 liver tumors 12.5%.  Ref: 12893427 Cancer Lett, 2003
- This inhibition was associated with decreased beta catenin  [ **CTNNA1** ]   [ **CTNNB1** ]  Tcf Lef dependent transcription, lack of multinucleated myotube formation, and expression of the muscle specific proteins.  Ref: 12878481 J Mol Cell Cardiol, 2003
- Lastly, stabilization of beta catenin  [ **CTNNA1** ]   [ **CTNNB1** ]  resulted in the retention of differentiated epithelium upon involution and altered activities of several proteases in transdifferentiated mammary epithelium.Oncogene 2003 2 594 4610.  Ref: 12881717 Oncogene, 2003
- TGF beta1 mediated MAP kinase activation also controls Wnt 7a gene expression and Wnt mediated signaling through the intracellular beta catenin  [ **CTNNA1** ]   [ **CTNNB1** ]  TCF pathway, which likely regulates N cadherin expression and subsequent N cadherin mediated cell adhesion complexes during the early steps of MPC chondrogenesis.  Ref: 12893825 J Biol ChemJ Biol Chem, 2003
- N and C Terminal Domains of beta Catenin  [ **CTNNA1** ]   [ **CTNNB1** ] , Respectively, Are Required to Initiate and Shape Axon Arbors of Retinal Ganglion Cells In Vivo.  Ref: 12878698 J NeurosciJ Neurosci, 2003
- Moreover, xHMG2L1 inhibited beta catenin  [ **CTNNA1** ]   [ **CTNNB1** ]  stimulated transcriptional activity in mammalian cells.  Ref: 12875653 Genes CellsGenes Cells, 2003
- In support of this we show that separation of thymocytes from Wnt producing epithelial cells and the thymic microenvironment, triggers beta catenin  [ **CTNNA1** ]   [ **CTNNB1** ]  phosphorylation and degradation in thymocytes.  Ref: 12884861 Eur J Immunol, 2003
- Conversely, siRNA mediated knockdown of endogenous TIP 1 slightly increased endogenous beta catenin  [ **CTNNA1** ]   [ **CTNNB1** ]  transactivation function.  Ref: 12874278 J Biol ChemJ Biol Chem, 2003
- Western blotting was used to determine polypeptide size and expression of E cadherin and beta catenin  [ **CTNNA1** ]   [ **CTNNB1** ]  in Wilms s tumours compared with normal kidney.  Ref: 12890743 Mol Pathol, 2003
- Thus, sustained exposure to Wnt in the context of an intact stromal microenvironment is necessary for stabilization of beta catenin  [ **CTNNA1** ]   [ **CTNNB1** ]  mediated signaling in thymocytes.  Ref: 12884861 Eur J Immunol, 2003
- Moreover, it is likely increased cyclin D1 expression results at least in part from Catnb mutation, beta catenin  [ **CTNNA1** ]   [ **CTNNB1** ]  accumulation, and increased Wnt signaling.  Ref: 12878043 Toxicol Appl Pharmacol, 2003
- Identification of genes differentially expressed in mouse mammary epithelium transformed by an activated beta catenin  [ **CTNNA1** ]   [ **CTNNB1** ] .  Ref: 12881717 Oncogene, 2003
- beta catenin  [ **CTNNA1** ]   [ **CTNNB1** ]  is one of the essential components of this specification.  Ref: 12885771 J Biol ChemJ Biol Chem, 2003
- OBJECTIVE To study the relationship between the abnormal expression of beta catenin  [ **CTNNA1** ]   [ **CTNNB1** ]  beta cat and the high expressions of cyclin D1 and c myc and the occurance, proliferation, infiltration, metastasis and prognosis of pancreatic cancer, and to provide rational basis for the clinical diagosis and treatment.  Ref: 12882690 Zhonghua Bing Li Xue Za Zhi, 2003
- CONCLUSIONS Immunostaining of beta catenin  [ **CTNNA1** ]   [ **CTNNB1** ]  is a useful marker for differentiating between ovarian mucinous carcinoma and metastatic colorectal adenocarcinoma.  Ref: 12877730 Histopathology, 2003
- The distribution of plakoglobin and beta catenin  [ **CTNNA1** ]   [ **CTNNB1** ]  was determined by immunofluorescence, western blot analysis, pulse chase radiolabeling, and biochemical subcellular fractionation.  Ref: 12880414 J Invest Dermatol, 2003
- Cellular retinol binding protein 1 in hepatocellular carcinoma correlates with beta catenin  [ **CTNNA1** ]   [ **CTNNB1** ] , Ki 67 index, and patient survival.  Ref: 12883492 HepatologyHepatology, 2003
- Distribution of cell cell adhesion molecules of E cadherin EC and beta catenin  [ **CTNNA1** ]   [ **CTNNB1** ]  BC infiltrating breast carcinoma BC and markers of epithelium, basal membrane has been studied by immunofluorescence in 50 infiltrating BC tissue samples including 17 ductal carcinomas, 23 lobular carcinomas and 10 combined carcinomas.  Ref: 12879601 Arkh PatolArkh Patol, 2003
- The disorganization of E cadherin catenin  [ **CTNNA1** ]   [ **CTNNB1** ]  complexes and the overexpression of matrix metalloproteinases MMPs are frequently involved in the capacity of epithelial cells to acquire an invasive phenotype.  Ref: 12875984 Am J Pathol, 2003
- This differentiation was preceded by nuclear accumulation of beta catenin  [ **CTNNA1** ]   [ **CTNNB1** ] , which was associated with increased Tcf Lef dependent transcription.  Ref: 12878481 J Mol Cell Cardiol, 2003
- CONCLUSIONS Compared with primary tumours, metastatic tumours showed lower expression of E cadherin and gamma catenin  [ **CTNNA1** ]   [ **CTNNB1** ] , with nuclear staining for beta catenin  [ **CTNNA1** ]   [ **CTNNB1** ] .  Ref: 12890743 Mol Pathol, 2003
- Nuclear localization of beta catenin  [ **CTNNA1** ]   [ **CTNNB1** ]  was found in 83% 19 23 of metastatic colorectal cancers and 9% 4 43 of ovarian mucinous carcinomas.  Ref: 12877730 Histopathology, 2003
- Reduced expression of E cadherin, which interacts with beta catenin  [ **CTNNA1** ]   [ **CTNNB1** ]  at the membrane, was observed in some tumors, but this did not correlate with Catnb mutation.  Ref: 12878043 Toxicol Appl Pharmacol, 2003
- A deletion mutant betacatDeltaARM consists of the N and C terminal domains of wild type beta catenin  [ **CTNNA1** ]   [ **CTNNB1** ]  that contain, respectively, alpha catenin  [ **CTNNA1** ]   [ **CTNNB1** ]  and postsynaptic density 95 PSD 95 discs large Dlg zona occludens 1 ZO 1 PDZ binding sites but lacks the central armadillo repeat region that binds cadherins and other proteins.  Ref: 12878698 J NeurosciJ Neurosci, 2003
- Primary and metastatic tumours showed moderate to high beta catenin  [ **CTNNA1** ]   [ **CTNNB1** ]  expression in blastemal and epithelial cells, with predominantly membranous and cytoplasmic staining.  Ref: 12890743 Mol Pathol, 2003
- The decrease of beta catenin  [ **CTNNA1** ]   [ **CTNNB1** ]  transcriptional activity in E cadherin transfected clones was demonstrated using the TOP FLASH reporter construct.  Ref: 12875984 Am J Pathol, 2003
- AIMS To investigate whether localization of beta catenin  [ **CTNNA1** ]   [ **CTNNB1** ]  is helpful in differentiating primary ovarian mucinous carcinoma and colorectal adenocarcinoma metastatic to the ovary.  Ref: 12877730 Histopathology, 2003
- The decrease of VE cadherin and beta catenin  [ **CTNNA1** ]   [ **CTNNB1** ]  in Triton insoluble fractions confirmed that the association of adherens junctions with actin cytoskeleton was altered during apoptosis.  Ref: 12878166 Exp Cell Res, 2003
- Higher amounts of 92 kDa beta catenin  [ **CTNNA1** ]   [ **CTNNB1** ]  were detected in tumours than in normal kidney.  Ref: 12890743 Mol Pathol, 2003
- These data suggest that evaluation of the immunohistochemical expression of E cadherin, alpha catenin  [ **CTNNA1** ]   [ **CTNNB1** ]  and beta catenin  [ **CTNNA1** ]   [ **CTNNB1** ]  is extremely valuable for the diagnosis of metastatic occurrence.British Journal of Cancer 2003 8 57 563.  Ref: 12888830 Br J Cancer, 2003
- Glomerular crescents predominantly express cadherin catenin  [ **CTNNA1** ]   [ **CTNNB1** ]  complex in pauci immune type crescentic glomerulonephritis.  Ref: 12877733 HistopathologyHistopathology, 2003
- In the metastatic process, reduction in cell to cell adhesion including E cadherin catenin  [ **CTNNA1** ]   [ **CTNNB1** ]  cell adhesion complex is an essential step.  Ref: 12888830 Br J Cancer, 2003
- Mutation analysis of  **CTNNB1**  beta catenin  [ **CTNNA1** ]   [ **CTNNB1** ]  and AXIN1, the components of Wnt pathway, in cervical carcinomas.  Ref: 12883680 Oncol RepOncol Rep, 2003
- Expression of the N and C terminal domains of beta catenin  [ **CTNNA1** ]   [ **CTNNB1** ]  separately in RGCs resulted in segregation of these two phenotypes.  Ref: 12878698 J NeurosciJ Neurosci, 2003
- We conclude that the capacity of lithium to overcome the inhibitory effects of serum and to induce the differentiation of H9c2 cardiomyoblasts is mediated, in part, by the stabilization and nuclear translocation of beta catenin  [ **CTNNA1** ]   [ **CTNNB1** ]  in a PI3 kinase dependent but Akt independent manner.  Ref: 12878481 J Mol Cell Cardiol, 2003
- Eight adult cases with myeloperoxidase anti neutrophil cytoplasmic autoantibodies MPO ANCA related pauci immune type crescentic glomerulonephritis were examined on immunofluorescence microscopy with anti pan cadherin, p120 catenin  [ **CTNNA1** ]   [ **CTNNB1** ] , and beta catenin  [ **CTNNA1** ]   [ **CTNNB1** ]  antibodies.  Ref: 12877733 HistopathologyHistopathology, 2003
- Expression of the epidermal growth factor receptor, which may have a role in beta catenin  [ **CTNNA1** ]   [ **CTNNB1** ]  tyrosine phosphorylation, was lower in some tumors than in normal tissue depending on chemical treatment.  Ref: 12878043 Toxicol Appl Pharmacol, 2003
- In order to identify novel binding partners of beta catenin  [ **CTNNA1** ]   [ **CTNNB1** ]  that may control its transcriptional activity we performed a mammalian two hybrid screen and isolated the Tax interacting protein TIP 1.  Ref: 12874278 J Biol ChemJ Biol Chem, 2003
- Expression of non destructible beta catenin  [ **CTNNA1** ]   [ **CTNNB1** ]  mutants increased survivin expression and protected against ultraviolet B induced apoptosis.Interpretation Stimulation of survivin expression by TCF beta catenin  [ **CTNNA1** ]   [ **CTNNB1** ]  might impose a stem cell like phenotype to colonic crypt epithelium coupling enhanced cell proliferation with resistance to apoptosis, and contribute to the molecular pathogenesis of colorectal cancer.  Ref: 12885482 Lancet, 2003
- Inhibition of PI3 kinase by LY294002 and over expression of dominant negative PI3 kinase caused a marked reduction in beta catenin  [ **CTNNA1** ]   [ **CTNNB1** ]  levels.  Ref: 12878481 J Mol Cell Cardiol, 2003
- Activation of Wnt signalling through mutations of APC or beta catenin  [ **CTNNA1** ]   [ **CTNNB1** ]  is a key event in the development of colorectal cancer.  Ref: 12877730 Histopathology, 2003
- In this chapter, we will review the current status of dynamic glycosylation phosphorylation of several important regulatory proteins including c Myc, estrogen receptors, Sp1, endothelial nitric oxide synthase, and beta catenin  [ **CTNNA1** ]   [ **CTNNB1** ] .  Ref: 12882516 Prog Nucleic Acid Res Mol Biol, 2003
- Although nuclear localization of beta catenin  [ **CTNNA1** ]   [ **CTNNB1** ]  was occasionally seen in non mucinous carcinoma of the ovary and metastatic adenocarcinoma from other organs, such tumours were usually distinguishable by their clinicopathological picture and rarely raised diagnostic problems.  Ref: 12877730 Histopathology, 2003
- Axin is a multidomain protein that plays a critical role in Wnt signaling, serving as a scaffold for down regulation of b catenin  [ **CTNNA1** ]   [ **CTNNB1** ] .  Ref: 12878610 J Biol ChemJ Biol Chem, 2003
- AIMS To evaluate E cadherin, beta catenin  [ **CTNNA1** ]   [ **CTNNB1** ] , gamma catenin  [ **CTNNA1** ]   [ **CTNNB1** ] , and ezrin expression and its relation to histopathological features of primary and metastatic Wilms s tumours.  Ref: 12890743 Mol Pathol, 2003
- We used deletion mutants to study beta catenin  [ **CTNNA1** ]   [ **CTNNB1** ]  function in axon arborization of retinal ganglion cells RGCs in live Xenopus laevis tadpoles.  Ref: 12878698 J NeurosciJ Neurosci, 2003
- Sections were immunostained with antibodies to beta catenin  [ **CTNNA1** ]   [ **CTNNB1** ] , cytokeratin CK 7, CK20 and carcinoembryonic antigen CEA.  Ref: 12877730 Histopathology, 2003
- These changes were related to a decrease in the expression of the adhesion molecules, E cadherin and beta catenin  [ **CTNNA1** ]   [ **CTNNB1** ] , and to the distribution and reactivity of actin microfilaments to TRITC phalloidin.  Ref: 12888906 Int J Oncol, 2003
- In this study, we screened liver tumors induced by 2 amino dimethylimidazo f quinoline MeIQ in CDF 1 mice for beta catenin  [ **CTNNA1** ]   [ **CTNNB1** ]  and APC mutations and other genetic alterations shown to occur in human hepatocellular carcinomas HCC, including mutations in the p53 and H ras genes, c myc amplification and E cadherin promoter methylation.  Ref: 12893427 Cancer Lett, 2003
- There was a close correlation between nuclear CRBP 1 inclusions and nuclear beta catenin  [ **CTNNA1** ]   [ **CTNNB1** ]  staining in HCCs P =.008, suggesting a cross talk between CRBP 1 and the Wnt wingless signal transduction pathway.  Ref: 12883492 HepatologyHepatology, 2003
- Differential expression of E cadherin and beta catenin  [ **CTNNA1** ]   [ **CTNNB1** ]  in primary and metastatic Wilms s tumours.  Ref: 12890743 Mol Pathol, 2003
